# Multivariate prediction of pain perception based on pre-stimulus activity

**DOI:** 10.1038/s41598-022-07208-1

**Published:** 2022-02-25

**Authors:** Philipp Taesler, Michael Rose

**Affiliations:** Department of Systems Neuroscience, University Medical Center Hamburg-Eppendorf, Martinistr. 52, Building W34, 20248 Hamburg, Germany

**Keywords:** Neuroscience, Physiology, Psychology

## Abstract

The perception of pain is modulated by different processes such as, for example, expectations and attention regarding the upcoming stimulus. Such processes are initiated prior to the actual stimulus and are reflected in ongoing brain activity. Different processes that are by definition also complex in itself are reflected in pre-stimulus activity and therefore the detection of this activity pattern should benefit from a multivariate approach. To identify specific pre-stimulus EEG activity patterns related to subsequent pain perception in humans, we contrasted painful with non-painful sensations delivered at the individual threshold level during EEG measurements. The results of the multivariate EEG analysis revealed a high level of accuracy (group mean 68%) in predicting the pain categorization solely based on pre-stimulus activity. In particular, fronto-central regions and activity in the higher gamma band (60:120 Hz) were of maximal importance for classification. Additional analyses supported the specific role of the pattern of high gamma band activity prior to the stimulus for predicting the behavioral outcome and demonstrated that the informational value embedded in the pre-stimulus activity is nearly as informative as the post-stimulus processing and reflects a specific preparatory state. Further, a close relation between pre- and post-stimulus processing in the high gamma band was observed. These findings support the important role of a multivariate cognitive state prior to stimulus appearance for the emergence of the subjective perception of pain and the functional role of widespread high gamma band activity.

## Introduction

The subjective feeling of pain is a highly multivariate concept^1^. The processing of a pain stimulus depends on the integration of several neural processing stages in multiple involved areas^2,3^ and the resulting pain percept is best explained by including activity from all areas of the network. Nociceptive processing have been related to a complex network that include somatosensory area (S1 and S2), Insular Cortex (IC), Anterior Cingulate Cortex, Prefrontal Cortex and the Thalamus^4^. The interaction within this network is in some parts overlapping with the processing of somatosensory, non-painful stimulation, which makes it difficult to disentangle activations within experimental paradigms^5,6^. Most of the network nodes have also been shown to be part of other, non-pain related networks, such as salience^7–9^ and interoception^10^. By using multivariate analyses of the neurologic signature of this network assessed by fMRI, a high sensitivity and specificity could be achieved in differentiating pain and non-painful sensation^3,11^. Also for EEG data it has been shown that a mulitvariate approach is able to predict the individual pain sensitivity with a high degree of accuracy (83%) and can detect the multidimensional nature of pain processing^12^.

The perception of pain is a subjective process and cannot solely be linked to stimulus intensity. The subjective experience of constant noxious stimulation also shows substantial intraindividual variability^13,14^. A single stimulus intensity can be experienced as painful, aversive, or even non-painful. Which sensation is experienced depends on the current cognitive state^15,16^. This effect can be assumed to be stronger in situations where stimuli around the individual pain threshold are presented. Many additional factors such as attention or expectation also influence whether a given stimulation is ultimately perceived as painful^17,18^. Also, spinal-level influences on signal transduction have been demonstrated e.g. for the placebo effect^19–21^. Many of such modulatory processes as expectancy or anxiety occur constantly prior to the actual stimulus input and therefore should be reflected in ongoing neural activity. The relevance of a prestimulus state of a neuronal assembly and subsequent stimulus processing has been demonstrated for various cognitive processes such as attention^22^, perception^23,24^ and memory^25^ and a functional relation was shown by manipulating this activity^26,27^. The importance of multivariate decoding has been recently also shown for object based attention^28^.

However, the neuronal activity within the anticipation phase during pain processing was examined only in very few studies. For the examination of the placebo effect on pain processing, increased activity of the DLPFC and OFC during the anticipation phase was reported^29^. Further, enhanced functional coupling of the anterior insula with the brainstem prior to the stimulus was related to the feeling of pain^30,31^. The relevance of a distributed network for consecutive pain processing was also demonstrated in an fMRI study that showed that activity in the default-mode network predict the subsequent magnitude of pain ratings^32^.

Further, it could be shown that a combination of alpha and gamma band prestimulus activity can predict the perception of nociceptive stimuli^33^. In two previous studies we have examined oscillatory activity in the pre-stimulus interval for a constant stimulus at the individual pain threshold^34,35^. For trials rated as painful, significant increases in the theta- (3–7 Hz) and gamma- (28–32 Hz) band before stimulus onset were observed. Further, we could demonstrate that a modulation of the theta-band activity by neurofeedback affects the differentiation of the consecutive stimulus with respect to pain^36^, indicating a functional role of pre-stimulus activity for pain processing. However, since it can be assumed that different networked processes produce widespread activity which reflects the modulatory cognitive state, the estimation of the functional relevance of pre-stimulus activity should benefit from a multivariate approach. Such an analysis should include data from different topographical areas and different frequency bands. With such an approach, the predictive value of an ongoing cognitive state for the behavioral outcome can be estimated both on the individual as well as the group level. Further, by comparing the prediction power for pre- and post-stimulus neuronal activity, the influence of pre-stimulus activity on subsequent perception can be estimated. Based on previous results it can be hypothesized that a multivariate approach within the prestimulus time period that included the gamma band activity and is based on different locations will predict the subsequent perception.

In general, the high dimensionality of EEG data (time, location and frequency) should be reduced for the classification procedure. The processes in the pre-stimulus period are not time locked to a specific event and can be assumed to reflect cognitive states rather than specific stimulus processing. This also means, that the time domain is less important for the present analysis, since specific activity distribution across location and frequency can be assumed to reflect the multivariate nature of the pre-stimulus state. Therefore, the present study includes multivariate analyses that classify the behavioral outcome (‘pain’ vs ‘no pain’) using the features “location” and “frequency” within the pre-stimulus period. As a control, the same analyses are used on the post-stimulus processing stage. To assess the combined “location × frequency”-pattern in the pre-stimulus period predicting the behavioral outcome of pain, we used a linear support vector machine (SVM), permitting a direct interpretation of the weight vector as class separating information. This allows us to classify the behavioral outcome of “pain”- vs. “no-pain” for the subsequent stimulus based on pre-stimulus oscillatory activity both on individual- as well as group level.

## Materials and methods

### Participants

Datasets from 33 healthy right handed participants were analyzed. One group was stimulated at the left hand (n = 20, 9 female, mean age 26.9 ± 6.9 years) and one group at the right hand (n = 13, 6 female, mean age 25.2 ± 7.3 years). Univariate analyses of subgroups from both experiments were previously published^34,35^. All participants gave written consent and received a compensation of 15 EUR per hour for their participation. The studies were approved by the local ethics committee (PV4509).

### Experimental procedure

Pain stimuli were delivered by applying electrical current to the abductor/flexor pollicis brevis of the left hand using a DS7A Peripheral Stimulator (Digitimer Ltd., Hertfordshire, UK). The stimulation was triggered using an experimental script in Matlab (R2009b; MathWorks, Natick, USA). The pulse length was constant at 2 ms, while the current was adjusted manually by the experimenter according to the value displayed by the script. Participants were asked to direct their attention to a freely chosen location on their body and keep it there constantly throughout the experiment.

The first block of 40 trials was used to determine the 50% pain threshold. Stimulation intensities were chosen according to values suggested by the QUEST algorithm^37^. This algorithm fits a psychophysical function by probing stimulus intensities and is optimized for the maximum likelihood estimation of a threshold. This method is well suited to efficiently determine the 50% threshold, located at the midpoint of the psychophysical function^38^. Participants were led to believe that the stimulus intensity would be chosen at random by the computer. They were asked to rate each stimulation using a mouse on a visual analog scale (VAS) after a 0.25 s delay. The scale ranged from “no sensation” (0) to “most extreme pain” (100), the center of the scale (50) representing the transition point between a strong sensation and painful stimulation. Regarding the classification into “painful” and “not painful” our experiment resembled a two alternative forced choice task, since the central point of the scale could not be selected. Two separate thresholding runs of 20 trials each were randomly interleaved to prevent participants from adjusting to the procedure. One thresholding run started from an arbitrarily chosen “high” intensity, while the other started out from a “low” intensity, to increase the chance of convergence. The individual threshold intensity for each participant was defined as the mean of the two resulting QUEST estimates.

Stimulus intensity was subsequently kept constant at threshold level for the remaining trial blocks. Participants were not made aware of this fact, but asked to keep rating the stimuli on the VAS as they had done so far. Dependent on the data quality assessed by on-line monitoring, participants then completed an additional four to six blocks of 30 trials each. This allowed us to directly compare painful and non-painful sensations within participants at constant stimulus intensity^39^.

On each trial participants were asked to keep their eyes on a fixation cross presented at the center of the screen. After a random waiting period of 3–5 s the stimulus was delivered. The VAS was presented onscreen 250 ms later. Participants rated the stimulus at their own pace. Following the rating, the fixation cross was shown and the next waiting period began. Visual material (fixation cross, instructions, rating scale) was rendered using the Psychophysics Toolbox version 3^40–42^ and displayed on a 23-inch TFT-Screen (SyncMaster P2370; Samsung, Seoul, South Korea) positioned centrally at about 1.1 m in front of the participant.

The experiment lasted approximately 45 min and participants were offered to take short breaks in between blocks. After finishing the experiment the true nature of the stimulation was revealed to the participants by the experimenter.

### Electroencephalographic (EEG) recordings

EEG data was acquired using a 64 channel Ag/AgCl active electrode system (ActiCap64; BrainProducts, Gilching, Germany) placed according to the extended 10–20 system^43^. 60 electrodes were used on the most central scalp positions. For off-line artifact removal a bidirectional, bipolar electrooculogram (EOG) was recorded using the remaining four electrodes. The bipolar EOG electrode pairs were placed above and below the left eye as well as on the lateral ends of the bicanthal plane. FCz was used as reference electrode for data recording, the ground electrode was placed at position Iz. The signal was digitized at a sampling rate of 250 Hz and high-pass filtered with a cutoff of 0.5 Hz at recording. All impedances were kept below 20 kΩ.

### Data analysis

EEG data were analyzed using the FieldTrip toolbox^44^. For each subject data were epoched into 2 s trials from − 2000 ms to 2000 ms around the stimulus onset. For artifact rejection a 2000 ms padding was added before and after the trials. To assist with visual artefact rejection, an automatic artifact tagging was then performed on the data, calculating trial based z-scores for EOG artifacts (1–15 Hz), muscle artifacts (100–120 Hz) and peaks in absolute difference between subsequent samples. Trials were then screened for unusual deviations in their maximum z-scores by the experimenter and an individual threshold for each dataset was chosen. All trials were additionally subjected to a full visual scan of the raw data. Epochs contaminated by artifacts were removed from the dataset in their entirety. The remaining trials for each subject were split into a “pain” and “no-pain” condition according to the VAS ratings (> 50 as pain). This was done by sorting the trials by rating in ascending order and selecting a matching number of trials from each end of the list until no more trials were left in either one of the conditions. Therefore, an equal number of trials in each condition was used in the subsequent analyses (40 trials on average in each condition).

For pain and non-pain trials two different time periods were selected for further analyses. To avoid any influence of the stimulus processing, the data for the pre-stimulus period were selected in the time range from − 1500 to − 250 ms and for the poststimulus period from 0 to 1000 ms and separate data segments were calculated. To reduce the complexity dimensions of the data and due to the fact that a pre-stimulus is not time locked to a specific event the classifier was estimated across frequencies and electrodes by the estimation of the frequency spectra across the time period.

Data were transformed into the frequency domain using the multi-taper method^45,46^ separately for the pre- and the post-stimulus period from 4 to 120 Hz (dpss tapering using 9 taper, frequency smoothing 4 Hz). The resulting frequency spectra for each participant and condition were then used to predict the individual pain sensitivity mainly for the pre-stimulus period but also for the poststimulus period. For the multivariate analyses the Donders Machine Learning Toolbox (DMLT^47^) was used. A support vector machine (SVM) with a five-fold cross-validation was used for classification after a standardization of the data (subtraction of the mean and division by the standard deviation). The classifier was trained on all electrodes using single-trial frequency spectra. Prediction accuracy and significance (binomial test) of prediction was assessed for each subject. On the group level prediction accuracy was tested statistically against values from a permutation distribution. Therefore 3300 iterations were estimated based on randomly selected data of pain and non-pain trial (100 from each participant). This process yields a distribution of classification accuracy values that could be expected by chance and the original prediction accuracy is tested against this distribution. The permutation was done separately for each of the used SVM analyses (pre- and poststimulus and SVM based on pre-stimulus gamma band activity).

To compare the prediction weights across subjects, the individual classification weights were averaged across all subjects but separately for left and right hand stimulation.

## Results

### Behavioral data

The overall median rating was 50.5, suggesting that the QUEST algorithm was successful at identifying the individual threshold intensities. Mean rating for the non-pain trials was 40 (SD = 12) and for the pain trial 55.2 (SD = 6.4).

### Classification based on pre-stimulus activity

For both stimulation sites, the mean classification accuracy across all subjects only for pre-stimulus oscillatory activity across all frequencies (4:120 Hz) was significantly above chance (mean accuracy across both sites: 0.68, *p* = 0.006 based on the permutation distribution, left hand: 0.66; right hand 0.7, mean of permutation distribution 0.5025). Importantly, the individual results revealed a significant prediction in 25 out of 33 participants (subject level accuracies are observed in the range from 0.63 to 0.94), demonstrating that the pre-stimulus neural activity can predict the subjective feeling of pain.

**Figure 1:**
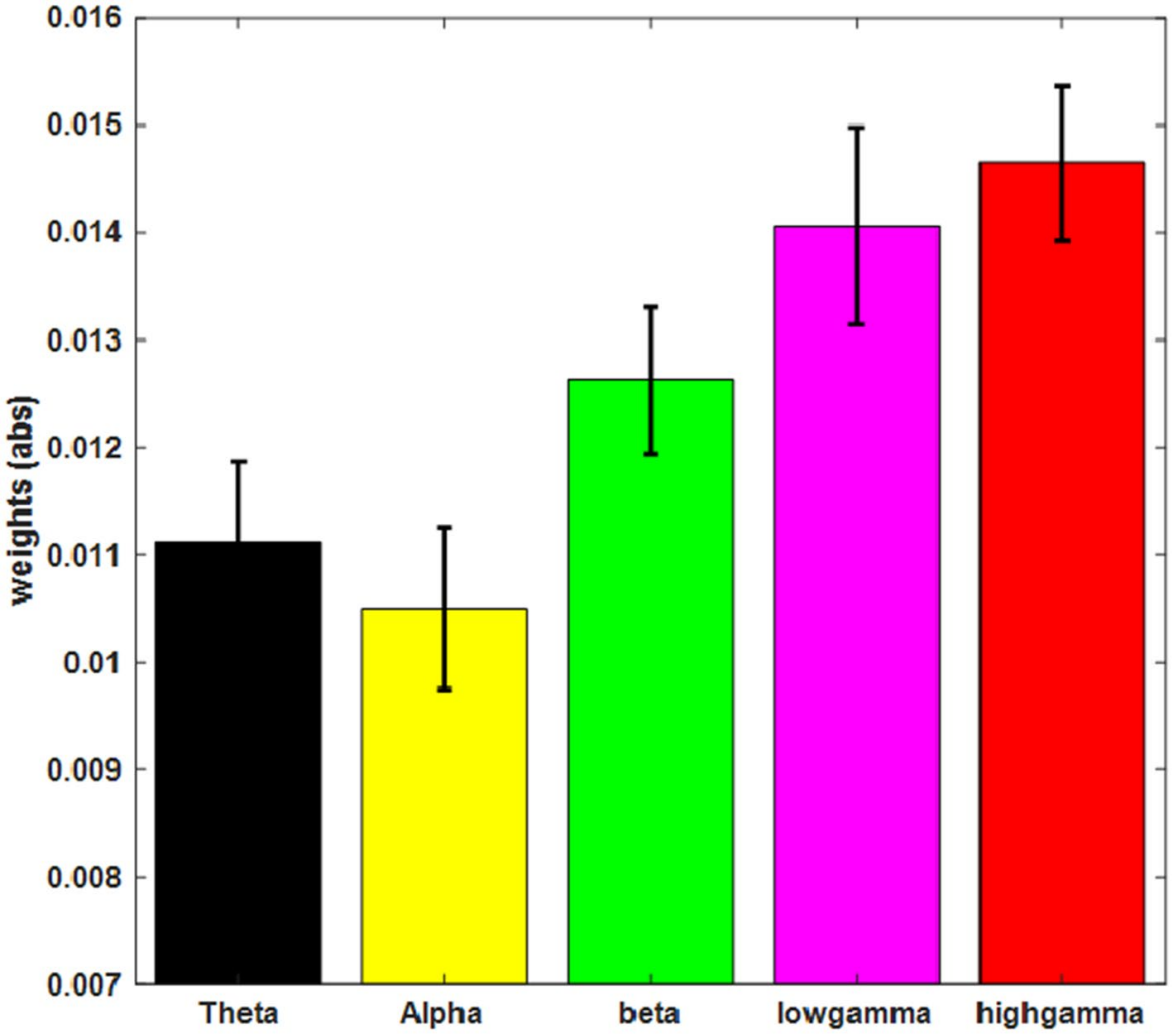
Distribution of maximal weights (abs) in the different frequency bands across the group of participants that could be reliably classified (n = 25). Nominal maximal weights were observed in the gamma band (low gamma: 30:59 Hz; high gamma: 60:120 Hz), but also theta- (4:7 Hz) and beta-band (13:24 Hz) contributed to the distributed prediction weights. Alpha (8:12) shows lower overall prediction weights.

To assess the main contributions of the different frequencies to the predictions the maximum weights (abs.) were estimated in each frequency band. As can be seen in Fig. 1 the maximal contributions to the prediction across all electrodes were observed in the gamma band, in particular for the high gamma band activity (above 45 Hz). From the 25 datasets with a reliable prediction in 22 the maximal prediction weight was observed in the gamma band underlining the importance of the gamma band in the pre-stimulus period for a successful classification. In three participants the maximal weights were observed in the theta-band.

**Figure 2:**
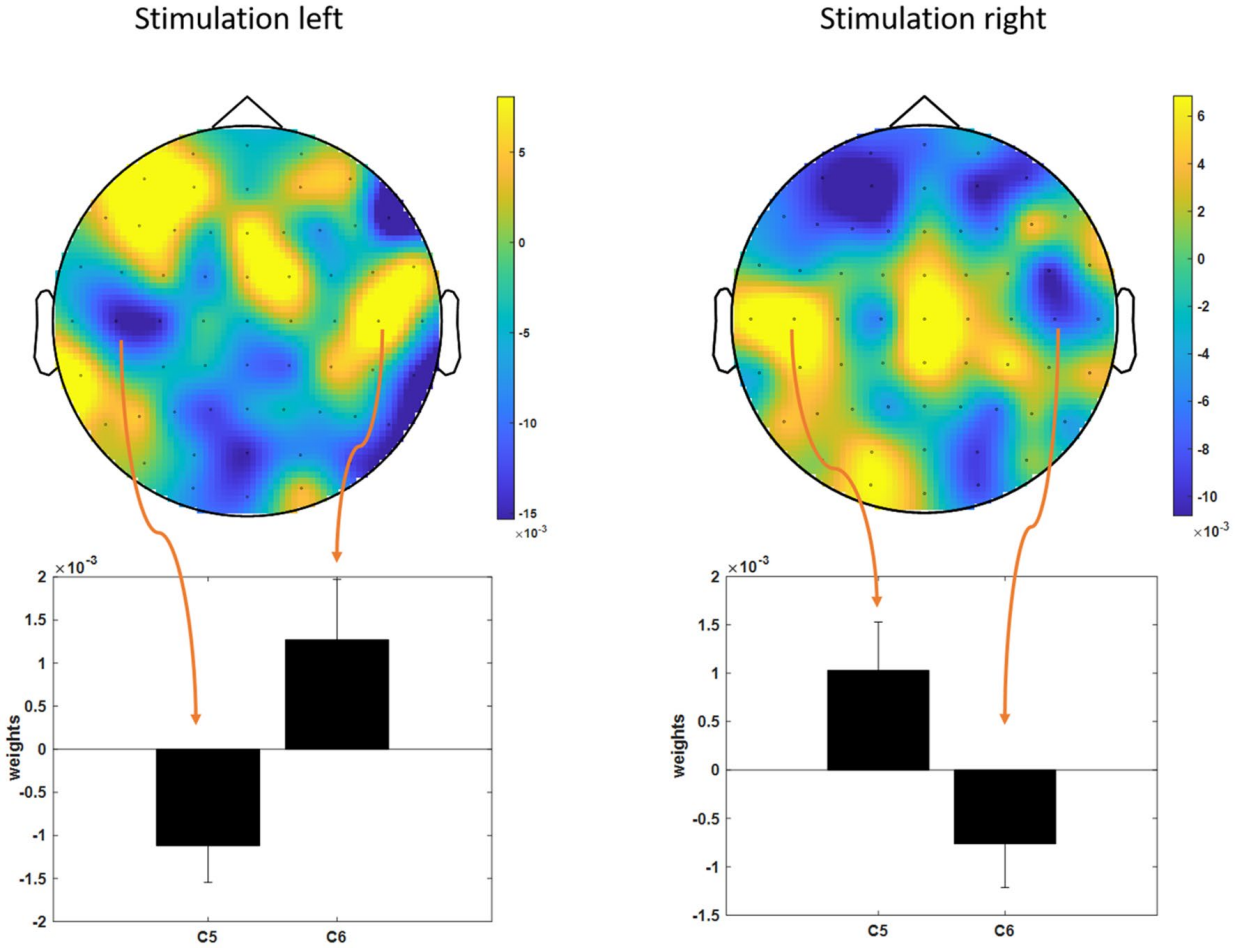
Topographical distribution of prediction weights in the high gamma band (60:120 Hz) showed a fronto-central focus. Over lateral central sites a lateralization was observed in correspondence to the stimulation site. For stimulation at the left hand the classification of painful trial resulted pre-stimulus in positive weights over the contralateral cortex and negative weights over the ipsilateral cortex and vice versa for right hand stimulations as shown for electrodes C5 and C6 below.

The topographical distribution of the high gamma band showed a frontal and fronto-central focus (see Fig. 2). Interestingly, the mean weights in the high gamma band showed a lateralization in correspondence with the stimulation site. For stimulation at the left hand the classification of painful trial resulted in positive weights over the contralateral cortex and negative weights over the ipsilateral cortex and vice versa for right hand stimulations (see Fig. 2 lower part). This effect was tested statistically by comparing the mean prediction weights of the electrodes C5 and C6 in the high gamma band using a rANOVA (factors stimulation site and electrode). The results demonstrated a clear interaction effect of stimulation sites and electrode (F(1,23) = 16.3, *p* < 0.001) indicating a specific preparatory state for pain processing that also reflected the anticipated site of stimulation.

To test the relevance of the high gamma band more directly we used the identical classification approach again limited to the frequency range 60:120 Hz. Again, the pain percept could be reliably classified by the pre-stimulus activity. The result showed a mean classification accuracy across all subjects across all electrodes of 0.68, which was significantly above chance (*p* = 0.007 on the permutation distribution) and nearly identical to the classification across all frequencies. Only one subject less could be classified significantly (24 instead of 25). This result indicates that the high gamma band is the most important factor to classify the resulting pain percept based on pre-stimulus activity. In comparison, using the theta-band, a classification accuracy of only 0.53 (n.s.) could be achieved. The pain rating could only be classified significantly in three participants using this data. Further, also by including all other frequencies without the gamma band no reliable classification could be achieved (accuracy for 4:24 Hz = 0.57; n.s.); by adding the low gamma band a reliable prediction could be achieved but the accuracy was lower (accuracy for 4:59 Hz = 0.65, *p* = 0.02) than for the high gamma band alone.

**Figure 3:**
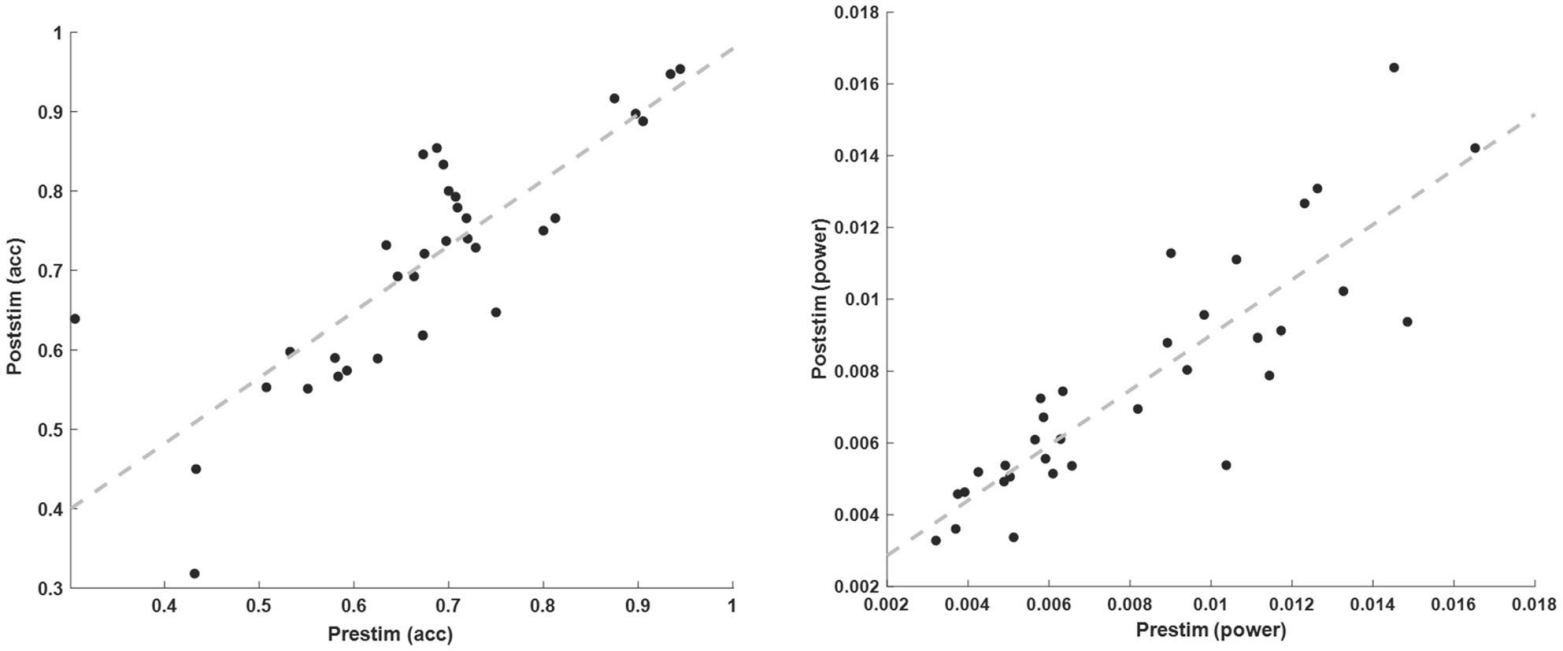
Relation between pre- and poststimulus time interval. Values for each of the 33 participants demonstrate a close correlation between both time periods (− 1.5:− 0.25 and 0:1 s) for the multivariate accuracies (left, r = 0.83) as well as for the high gamma band power extracted from the electrode FCz (right, r = 0.87).

To compare pre- and poststimulus contributions, the identical analysis was also performed in the poststimulus period (0:1 s). For both stimulation sites, the mean classification accuracy across all subjects was 0.71 (significantly above chance, *p* = 0.0045, based on the permutation distribution). Using this time interval the rating from 25 out of 33 participants could be correctly classified (each *p* < 0.05). However, a statistical test between the accuracies of pre- and poststimulus period showed no reliable difference (t(64) = 0.39, n.s.). From the seven participants that could not be predicted by the pre-stimulus period, four could also not be classified by the poststimulus activity. Furthermore, the accuracies from both time intervals were highly correlated (r = 0.83, *p* < 0.0001, see Fig. 3 left), demonstrating that the pre-stimulus activity affects the processing of the stimulus and the behavioral outcome to a reliable degree and/or contain nearly comparable amount of information to predict the behavioral outcome as the poststimulus processing.

To further evaluate the relation of pre-and poststimulus activity in the gamma band without a multivariate approach a correlation of high gamma band power (60:120 Hz) at electrode FCz was estimated between both time periods. Over the whole group of participants the individual mean values for pre- and poststimulus gamma band power resulted in a clear correlation of 0.87 (*p* < 0.001) (see Fig. 3 right).

**Figure 4:**
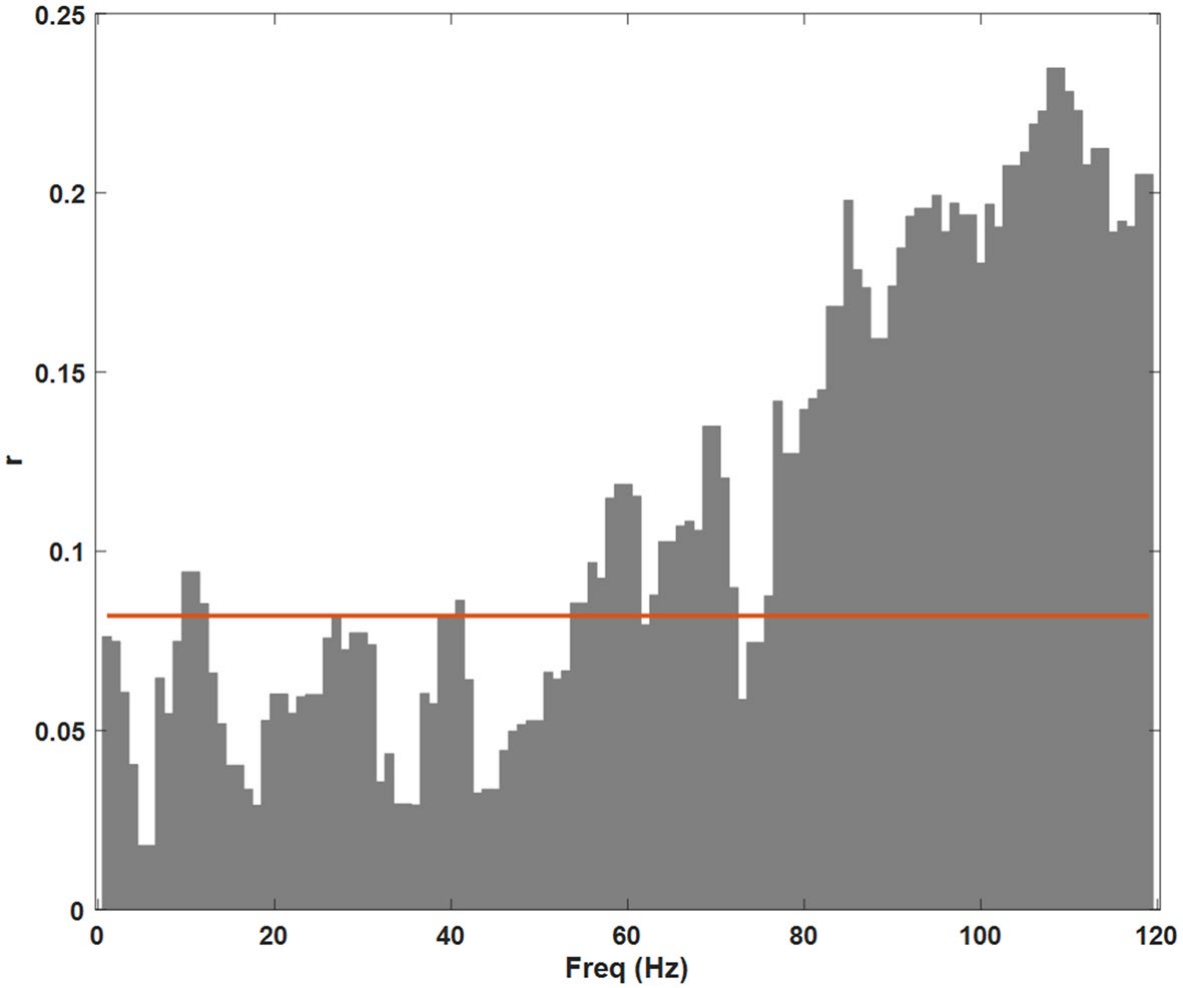
Correlation of pre- and poststimulus activity for each frequency at electrode FCz. The red bar indicated the significance level (Bonferroni corrected). It can be seen that a clear relation of pre- and poststimulus activity is nearly restricted to the gamma band range.

To test for a more general relation of pre- and poststimulus activity we performed a correlation for all frequencies on a single frequency level (given a higher level of noise) at electrode FCz. As can be seen in Fig. 4 a significant correlation (Bonferroni corrected) was observed only in the gamma band mainly above 60 Hz (only 3 Frequencies outside the gamma band were significant: 9, 10 and 23 Hz). No differences between pain- and no pain trials were observed. This further indicate the importance of the gamma band for the initiation of a specific pain related cognitive states that is transmitted to the post-stimulus processing.

The obvious correlation of the pre- and poststimulus high gamma band activity may indicate that the induced cognitive state is more stable in time and spans across more trials. To test this assumptions an autocorrelation of the time series of high gamma band prestimulus activity across all trials was estimated for each participant (from lag 1 to lag 20) and was compared against the respective confidence interval. For lag 1 (from trial to trial) a reliable correlation was found in only 5 from 33 participants (mean across the whole group: 0.12) and for lag 2 only one participant showed a reliable relation.

## Discussion

The results from the multivariate analyses of the pre-stimulus period demonstrate a high level of accuracy for the classification of subjective pain ratings for the upcoming stimulus. For 25 out of 33 subjects a reliable classification can be achieved only based on pre-stimulus activity with an accuracy of 0.68 across the whole group. The overall classification accuracy based on the actual processing of the stimulus was nominally higher (0.71) but statistically not significant. The fact that four participants could not be classified correctly for both time periods indicates an even higher accuracy, since it can be speculated that the rating of this four participants may be more random and the behavioral indicator was not reliable. This results demonstrate the importance of pre-stimulus processing states for the processing of a consecutive stimulus with respect to the subjective feeling of pain and supported the view of pain as a highly multivariate concept^1^. A lot of information about the outcome of pain processing is already established prior to the actual stimulus and this cognitive state has a large influence of the development of the subjective feeling of pain.

The main contributions to the classification could be related to the gamma band activity, in particular to the high gamma band activity above 60 Hz. Interestingly, the weights of the high gamma band show a lateralization that reflect the different stimulation sites—indicating a highly specific preparatory state within this activity pattern. For the majority of participants the maximal weights are within this high-frequency band over frontal and fronto-central electrodes. Further, the accuracy of the SVM analyses limited to pre-stimulus gamma band activity is nearly identical to the classification across all frequencies demonstrating the importance of this frequency during the anticipation of an upcoming noxious stimulus. For poststimulus processing theta- and gamma-band activity are well described effects that are assumed to reflect different processes during pain related processing^48–50^. In particular, for activity in the high gamma band above 60 Hz a close relation has been reported to objective stimulus intensity as well as subjective feeling of pain^50,51^. It was concluded that theta responses are likely to reflect constant physiological and psychological traits of an individual, whereas gamma responses relate to short-term modulations of an individual's state^48^. The results from the present analyses of the power values at electrode FCz show that the evoked high gamma band response is highly correlated with the gamma band activity observed in the pre-stimulus phase, indicating that the cognitive state that modulates the individual pain feeling is also expressed in the gamma band activity and has a reliable influence on the stimulus processing. The comparison across all frequencies showed that this relation between pre- and poststimulus activity is clearly dominant in the frequency range above 60 Hz supporting the specific role of the gamma band. The estimation of the autocorrelation coefficients for the high gamma band prestimulus activity demonstrate that this state can be regarded as trial-specific and did not affects the next trial activity. Therefore, this state can be seen more as a variable cognitive rather than a constant mental state.

The observation that the accuracies from pre- and poststimulus time interval are highly correlated demonstrates that the pre-stimulus activity affects the processing of the stimulus and the behavioral outcome to a reliable degree. Furthermore, it shows, that a comparable amount of information to predict the behavioral outcome is contained in the pre-stimulus state and in post-stimulus processing. This may be related to the specific experimental setting with a stimulus at the pain threshold. In this setting, all pre-stimulus states may have a larger impact on the subjective feeling of pain and the behavioral outcome could already be determined to a large degree by the pre-stimulus states than for more salient stimulations above threshold. Pain processing has been formally conceptualized within a predictive coding framework, where the pain percept is critically determined by expectations and their modification through learning^52^. Here, expectations are formulated as top-down processes which affect pain processing via Bayesian integration. In this concept, a near threshold stimulus provides only little information and therefore can be affected to a large degree by pre-stimulus states like expectation. Therefore, this experimental setting is optimal for the examination of pre-stimulus modulatory effects but the results should be compared to above threshold stimuli. Further, to allow a comparison of pre- and post-stimulus processing the analysis approach in the present study was kept identical. For post-stimulus processing, analyzing the time-average is not optimal, since it was shown that specific theta- and gamma-band activity around 150–300 ms at electrode FCz can solely predict the individual pain sensitivity with an accuracy of 0.83 (Schulz et al., 2012). Both, the correlation of the multivariate accuracies as well as the correlation of the gamma band power values indicate a linear relation between pre- and poststimulus activity. However, a more complicated, non-additive interaction of pre-stimulus activity with the later processing stages has previously been demonstrated for other perceptual domains^23^.

Previous univariate analyses of an identical paradigm also showed effects within the theta- and gamma band^34,35^. In contrast, the theta-band was used to a smaller degree within the present multivariate approach, and the analysis limited to the theta-band showed no reliable classification performance. In contrast to univariate data analyses, the multivariate approach makes use of individual differences that cannot be detected by univariate analyses. The vast majority of participants showed maximal multivariate effects within the gamma band. However, the range within the gamma band shows a large variability regarding the exact frequency, and for three participants theta-band was most highly weighted for a correct classification. This variability can be used for individual multivariate analyses and in particular for pre-stimulus effects the individual differences may reflect variants in the cognitive state that modulates pain processing. The exact nature of this cognitive state and the different processes that are involved in the establishment of this modulatory network should be examined in more detail. For other domains like memory, it was demonstrated that the intention to encode the following stimulus into long term memory affects these preparatory pre-stimulus activations during encoding^53^. These findings indicate that the pre-stimulus effects are not purely related to randomly occurring fluctuations but rather reflect task specific preparatory states. The fact that the weights of the high gamma band in the present study are related to the stimulation site also indicated a specific preparatory state. In the context of pain, one specific process during the anticipatory period is threat in the anticipatory period that can affect pain processing and the effect could be related to activity in the anterior insular cortex^31^. In contrast, also more unspecific and spontaneous fluctuations can affect pain processing and have been integrated to the concept of a dynamic 'pain connectome' in the brain^54^. It is likely that a variety of such different processes are expressed in the pre-stimulus period simultaneously, and that the expression may also vary to a certain amount between different participants. This is a clear indicator for using multivariate methods to estimate the overall effect of prestimulus activity on pain processing.

Regarding the topographical findings, it is difficult to draw clear connections to neuroanatomical details. Imaging studies indicate the existence of multiple pathways that reflect the integration of expectation in pain-related processing. In particular, the DLPFC, ventromedial prefrontal cortex (VMPFC), lateral orbitofrontal cortex (LOFC), precuneus and temporoparietal junction (Geuter et al., 2017) are involved in creating the overall pain percept. In particular, the DLPFC has been associated with pain regulation and with the generation, maintenance, and manipulation of cognitive representations (Wager et al., 2004). Using rTMS in a heat-pain paradigm, the suppression of DLPFC function resulted in a reduced placebo effect, indicating a functional role of the DLPFC for the mediation of expectation based effects on pain processing (Krummenacher et al., 2010). The functional role of the DLPFC and gamma band activity for the mediation of expectation-based effects can be observed also in other cognitive domains like attention^55^.

However, the different processes that result in the multivariate concept of expectation or cognitive states are not fully understood and differentiated yet.

In summary, the present multivariate approach revealed an important role of the high gamma band to describe the widespread nature of the multivariate concept of a cognitive state that modulates pain processing.

## Data Availability

The datasets generated during and/or analysed during the current study are available from the corresponding author on reasonable request. We confirm that all methods were carried out in accordance with relevant guidelines and regulations and that all experimental protocols were approved by the ethics committee at the Hamburg Medical Association (PV4509). Informed consent was obtained from all subjects.
